# Trop2-targeted PET/CT imaging with ^68^Ga-MY6349 improves diagnostic accuracy and influences clinical management in breast cancer

**DOI:** 10.7150/thno.136704

**Published:** 2026-07-13

**Authors:** Lingyu Yu, Liang Zhao, Ru Zeng, Yonghui Su, Zhenyu Wu, Shan Yu, Yin Li, Hao Fu, Wei Guo, Yining Sun, Hannan Gao, Long Sun, Hua Wu, Feng Ye, Fan Wang, Haojun Chen

**Affiliations:** 1 Department of Nuclear Medicine and Minnan PET Center, Xiamen Key Laboratory of Radiopharmaceuticals, The First Affiliated Hospital of Xiamen University, School of Medicine, Xiamen University, Xiamen, China.; 2 Department of Medical Oncology, The First Affiliated Hospital of Xiamen University, School of Medicine, Xiamen University, Xiamen, China.; 3 Department of Breast Surgery, The First Affiliated Hospital of Xiamen University, School of Medicine, Xiamen University, Xiamen, China.; 4 State Key Laboratory of Biomacromolecules, Institute of Biophysics, Chinese Academy of Sciences, Beijing, China.; 5 Medical Isotopes Research Center and Department of Radiation Medicine, School of Basic Medical Sciences, Frontiers Science Center for Cancer Integrative Omics, International Cancer Institute, Peking University, Beijing, China.

**Keywords:** breast cancer, imaging-guided therapy, molecular imaging, PET/CT, Trop2

## Abstract

***Rationale:*** Trophoblast cell-surface antigen 2 (Trop2) is an essential therapeutic target in breast cancer, yet non-invasive methods for assessing its expression and predicting response to Trop2-directed antibody-drug conjugates (ADCs) remain limited. We aimed to evaluate the diagnostic accuracy of ^68^Ga-MY6349 positron emission tomography/computed tomography (PET/CT) in patients with breast cancer and assess its impact on clinical decision-making.

***Methods:*** We prospectively enrolled 73 patients with suspected or confirmed breast cancer who underwent both ^68^Ga-MY6349 and ^18^F-fluorodeoxyglucose (^18^F-FDG) PET/CT from December 2024 to April 2025. Lesion-based diagnostic performance was compared using histopathology and follow-up imaging as reference standards. Treatment decisions were recorded before and after ^68^Ga-MY6349 PET/CT. Serial ^68^Ga-MY6349 PET/CT was performed to assess the early metabolic response in three patients with triple-negative breast cancer receiving sacituzumab tirumotecan.

***Results:***^68^Ga-MY6349 PET/CT identified more malignant lesions (564 vs. 436) and fewer false positives (2 vs. 40) than ^18^F-FDG PET/CT. In the initial-staging cohort (n = 30), ^68^Ga-MY6349 PET/CT led to TNM stage upgrades in 6/30 patients (20%) and treatment modifications in 4/30. In the restaging cohort (n = 43), clinical management was altered in 5/43 patients (12%) owing to the identification of additional metastatic lesions or rectification of false-positive findings via ^68^Ga-MY6349 PET/CT. ^68^Ga-MY6349 PET/CT yielded higher overall tumor uptake (median maximum standardized uptake value (SUV_max_), 5.9 vs. 4.1; P < 0.001) and improved lesion conspicuity, particularly in lymph node and metastatic lesions. Exploratory analysis in three patients receiving Trop2-ADC therapy showed that early changes in ^68^Ga-MY6349 uptake after two treatment cycles were concordant with the subsequent clinical response.

***Conclusions:***^68^Ga-MY6349 PET/CT demonstrated superior diagnostic performance to ^18^F-FDG PET/CT in breast cancer staging and diagnosis, directly influencing therapeutic strategies. Preliminary findings from the triple-negative breast cancer cases suggest that early changes in SUV_max_ may be associated with the treatment response to Trop2-targeted ADC therapy, warranting further prospective validation in larger patient populations.

## Introduction

The rapid development of tumor-targeted therapies marked the era of precision medicine in cancer treatment 1. Through targeted therapy, patients benefit from streamlined and personalized treatment plans, which significantly improve cure and survival rates 2. The effectiveness of targeted therapy is often related to the expression levels of its molecular targets 3-5. However, owing to tumors’ temporal and spatial heterogeneity, current biopsy sampling for immunohistochemical analysis and genetic testing has limitations 6, 7. The accurate, non-invasive detection and quantification of molecular-target expression is essential in clinical medicine and an ongoing challenge. Nuclear medicine molecular imaging, with its *in vivo*, non-invasive, real-time, and quantitative features, offers potential solutions to these issues.

Trophoblast cell-surface antigen 2 (Trop2) is highly overexpressed in numerous epithelial malignancies and minimally expressed in normal adult tissues 8, 9. Its expression has been associated with poor prognosis in various cancers 10, 11. Hence, Trop2 is a promising target for both diagnostic imaging and therapeutic interventions. Sacituzumab govitecan, the first US Food and Drug Administration-approved Trop2-targeted antibody–drug conjugate (ADC), is indicated for patients with triple-negative breast cancer (TNBC) and those with hormone receptor-positive BC 12-14.

We previously developed a Trop2-targeted molecular imaging probe, ^68^Ga-MY6349, and conducted^ 68^Ga-MY6349 PET/CT in 15 cancer types, validating its clinical value 15. In this study, we focus on the application of ^68^Ga-MY6349 PET/CT in BC to evaluate its diagnostic accuracy and assess its impact on treatment decision-making.

## Methods

### Study Design and Patient Enrollment

This study was conducted in accordance with the principles of the Declaration of Helsinki (2024), was approved by the Ethics Committee of the First Affiliated Hospital of Xiamen University and the National Human Research Ethics Committee, and was registered at ClinicalTrials.gov (identifier: NCT07046702). Written informed consent was obtained from all participants prior to enrollment. The study was carried out at the First Affiliated Hospital of Xiamen University between December 2024 and April 2025.

We included (1) adult patients (> 18 years) with suspected, newly diagnosed, or previously treated BC, (2) those who had not received anticancer therapy in the 4 weeks prior to PET/CT imaging, and (3) those who provided written informed consent in accordance with the Institutional Review Board’s guidelines. The exclusion criteria comprised pregnancy, lack of informed consent, or a confirmed benign diagnosis at initial presentation.

Tumor characteristics, including size, number, and anatomical location, were documented. Additional clinical and biological data were collected for each patient, including age, sex, histological type, molecular subtype, and prior or ongoing treatments. Tumor-specimen immunohistochemistry (IHC) was performed to assess estrogen receptor, progesterone receptor, Ki-67, and human epidermal growth factor receptor 2 (HER2) expression. Tumors were classified into five molecular subtypes, i.e., luminal A, luminal B HER2-negative, luminal B HER2-positive, HER2-positive, and TNBC, based on the IHC results.

### PET/CT Imaging Acquisition Protocol

MY6349 precursor synthesis and radiolabeling with ^68^Ga were performed as previously described 15. Both FDG and ^68^Ga-MY6349 met a radiochemical purity threshold of > 95% before administration. All radiotracers underwent quality control according to institutional standards. ^68^Ga-MY6349 PET/CT was performed within a week of the ^18^F-FDG PET/CT scan. Additional acquisition and reconstruction parameters are detailed in the Supplementary Appendix.

### Image Review and Interpretation

PET/CT images were reviewed in the coronal, axial, and sagittal planes on an AW 4.7 by two board-certified nuclear medicine physicians (C.H. and W.H.), each with extensive experience in PET/CT interpretation. The readers were blinded to clinical information, pathological findings, and the results of the corresponding PET/CT examination performed using the alternative tracer. A lesion was considered PET-positive when the radiotracer uptake was higher than in the surrounding background upon visual examination; absence of abnormal uptake was defined as PET-negative 16. Any discrepancies were resolved by consensus. For lesions newly identified via ^68^Ga-MY6349 PET/CT, treatment decisions were made by a multidisciplinary team comprising specialists in nuclear medicine, medical oncology, radiation oncology, and breast surgery 16. Semiquantitative analysis included measurement of the maximum standardized uptake value (SUV_max_) and tumor-to-background ratio (TBR). Up to 10 lesions were evaluated for patients with metastatic disease, either all lesions if ≤ 10 or the 10 largest lesions if >10. Tumor staging was performed according to the American Joint Committee on Cancer, 8th edition, criteria.

All treatment-naïve patients underwent breast ultrasound as standard-of-care imaging in the 2 weeks prior to PET/CT. Lesions were classified according to the Breast Imaging Reporting and Data System (BI-RADS) ultrasound atlas 17. Nodules categorized as BI-RADS 4b or higher (4b and 4c correspond to malignancy likelihoods of 10%–50% and 50%–95%, respectively) were suspected as malignant.

### Reference Standards

Histopathological confirmation via surgical resection or biopsy served as the reference standard. All treatment-naïve patients (n = 33) were required to undergo tissue confirmation within 2 weeks of PET/CT imaging. In the absence of biopsy findings, PET/CT findings for restaging were verified through clinical follow-up of at least 6 months, supported by follow-up imaging studies, including ultrasound, CT, magnetic resonance imaging (MRI), PET/CT, and radionuclide bone scans, together with clinical assessment. Contrast-enhanced MRI served as the reference standard for brain lesions. Lacking histopathological confirmation, lesions were considered malignant upon showing progressive enlargement, newly developed structural abnormalities, or a typical metastatic distribution pattern. Benign lesions were classified as true-negative if there was no clinical evidence of disease progression and they remained stable or resolved during follow up.

### Statistical Analysis

Statistical analyses were performed using IBM SPSS Statistics for Windows (version 22.0; IBM Corp., Armonk, NY, USA). Normally distributed continuous variables are reported as means ± standard deviations, whereas non-normally distributed data are summarized as medians with Q1–Q3. Differences in paired SUV_max_ and TBR values between ^18^F-FDG and ^68^Ga-MY6349 PET/CT scans were assessed using the Wilcoxon signed-rank test. Group comparisons for continuous variables were conducted using the Mann–Whitney *U* test or independent-samples *t*-test, depending on data-distribution normality. A two-sided P-value < 0.05 was considered statistically significant.

## Results

### Patient Characteristics

**Figure 1:**
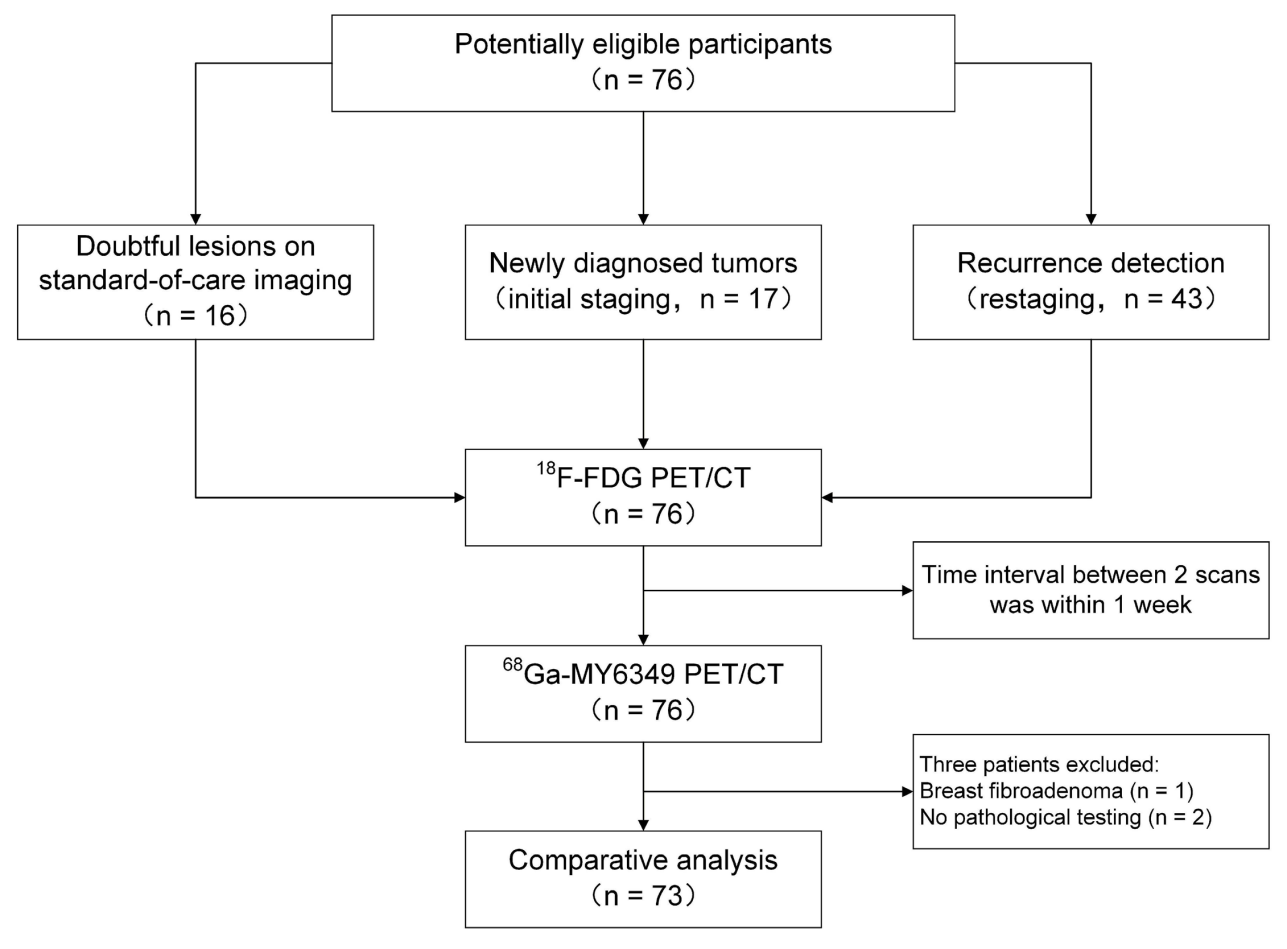
Flowchart illustrating the patient selection process. ^18^F-FDG: ^18^F-fluorodeoxyglucose; CT: computed tomography; PET: positron emission tomography.

From December 2024 to April 2025, 76 patients who underwent paired ^68^Ga-MY6349 and ^18^F-FDG PET/CT scans were prospectively enrolled for clinical evaluation. Among those, 16 were examined for suspected breast lesions, 17 were scanned for initial tumor staging, and 43 were examined for tumor recurrence or metastasis (restaging) detection. Three patients were excluded from the final analysis: one because of primary-malignancy absence and two because of lack of pathological confirmation. Eventually, 73 patients with pathologically confirmed BC were included in the study. The patient selection flowchart is presented in Figure 1. The median age of the patients was 52 years (range: 29–86 years). Based on molecular subtyping, patients were classified as: luminal A (n = 7), luminal B HER2-positive (n = 8), luminal B HER2-negative (n = 30), HER2-positive (n = 9), and TNBC (n = 19). Patient characteristics are summarized in Table 1.

### ^68^Ga-MY6349 PET/CT Exhibits Superior Tumor Uptake and Image Contrast to ^18^F-FDG PET/CT

Compared with ^18^F-FDG PET/CT, ^68^Ga-MY6349 PET/CT yielded a significantly higher tumor uptake (median [Q1-Q3] SUV_max_: 5.9 [3.6-8.6] vs. 4.1 [2.5-7.2], P < 0.001) and TBR (6.0 [3.6-9.6] vs. 3.4 [2.1-6.7], P < 0.001) when analyzed across all tumor types (Table 2), and improved lesion conspicuity, particularly in lymph node and metastatic lesions. Although the differences in tumor uptake between the tracers were not significant for breast lesions (including both primary and recurrent tumors), ^68^Ga-MY6349 PET/CT yielded significantly higher TBRs in these regions (6.6 [3.3-11.2] vs. 2.9 [1.3-4.8], P < 0.001). Similarly, the difference in SUV_max_ for brain metastases was not significant, but ^68^Ga-MY6349 showed no physiological uptake in normal brain tissues, resulting in a significantly higher TBR (6.1 [4.3-23.7] vs. 1.0 [0.8-1.0], P = 0.018). ^18^F-FDG PET/CT failed to detect 4/7 brain metastases, whereas ^68^Ga-MY6349 PET/CT missed none. Both tumor uptake and TBR were significantly greater with ^68^Ga-MY6349 PET/CT than with ^18^F-FDG PET/CT for lymph-node, bone, and visceral metastasis assessment. Specifically, the SUV_max_ for lymph node lesions was 5.6 (3.5–8.1) with ^68^Ga-MY6349 and 4.2 (2.4–7.0) with ^18^F-FDG (P = 0.002). Similarly, for bone and visceral metastases, SUV_max_ values were 6.3 (3.6–8.8) for ^68^Ga-MY6349 and 4.0 (2.6–6.8) for ^18^F-FDG (P < 0.001). Corresponding TBRs were higher with ^68^Ga-MY6349 than with ^18^F-FDG, with TBR values for lymph-node lesions at 7.8 (4.5–12.9) vs. 5.3 (3.0–9.6) (P < 0.001) and for bone and visceral metastases at 5.0 (2.6–7.1) vs. 3.0 (1.8–5.2; P < 0.001; Table 2). Notably, the superior tumor uptake and TBR with ^68^Ga-MY6349 were most pronounced in the luminal B HER2-negative and TNBC subtypes (Tables S1–S5).

### ^68^Ga-MY6349 PET/CT Yields Higher Diagnostic Accuracy for Primary and Metastatic BC Compared with ^18^F-FDG PET/CT

**Figure 2:**
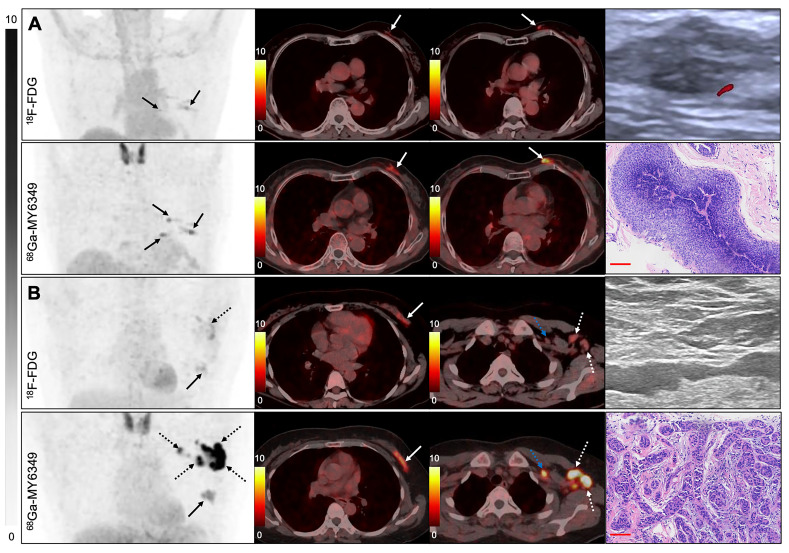
PET/CT and breast ultrasound features of suspected breast lesions and lymph node involvement. (**A**) A 70-year-old woman presented with a palpable breast nodule. ^18^F-FDG PET/CT revealed two irregular lesions in the left breast, whereas ^68^Ga-MY6349 PET/CT revealed four abnormal foci (solid arrows). Breast ultrasound demonstrated hypoechoic nodules classified as BI-RADS 4B. Histopathological examination confirmed a diagnosis of invasive ductal carcinoma. Scale bar: 100 μm. (**B**) A 51-year-old woman presented with axillary lymphadenopathy. Both ^18^F-FDG and ^68^Ga-MY6349 PET/CT identified a primary breast lesion (solid arrows). However, ^68^Ga-MY6349 PET/CT exhibited higher tracer uptake in axillary lymph nodes than ^18^F-FDG PET/CT (dashed arrows) and additionally revealed left supraclavicular lymph node involvement (blue dashed arrow), leading to an upgrade in nodal staging from N2 to N3. ^18^F-FDG:^ 18^F-fluorodeoxyglucose; BI-RADS: Breast Imaging Reporting and Data System; CT: computed tomography; PET: positron emission tomography.

Among the 30 patients with newly diagnosed BC, two presented with occult breast tumors, whereas seven had multifocal breast tumors. This multifocality included one patient with eight lesions, one with five, one with four, two with three, and two with two lesions. In total, the diagnostic performance analysis yielded 48 pathologically confirmed primary breast tumors. Of these, breast ultrasound detected 40 lesions (sensitivity: 83%), and ^18^F-FDG PET/CT detected 38 (sensitivity: 79%). ^68^Ga-MY6349 PET/CT demonstrated superior sensitivity, successfully identifying all 48 lesions (sensitivity: 100%; Figure 2A). Furthermore, when all breast lesions, including primary and recurrent tumors, were evaluated, ^68^Ga-MY6349 PET/CT consistently identified a greater number of true-positive lesions than ^18^F-FDG PET/CT, with totals of 59 and 50 lesions, respectively (Table 3).

^68^Ga-MY6349 PET/CT exhibited superior diagnostic performance than ^18^F-FDG PET/CT for regional and distant lymph-node metastasis evaluation. Specifically, ^68^Ga-MY6349 PET/CT identified 55 additional true-positive metastatic lymph nodes, but missed eight, compared with ^18^F-FDG PET/CT, with detection rates of 213/224 vs. 166/224, respectively (Figure 2B, Table 3). Notably, ^68^Ga-MY6349 PET/CT yielded no false-positive results, in stark contrast with the 36 false positives detected via ^18^F-FDG PET/CT. Figure S1 presents a representative case of false-positive ^18^F-FDG PET/CT results in the mediastinal and hilar lymph nodes, possibly attributable to the appearance of calcification and symmetrical distribution. A pulmonary lesion was histologically confirmed as comprising benign proliferating myofibroblasts with increased ^18^F-FDG uptake. Importantly, all these lesions were accurately identified as negative for metastases via ^68^Ga-MY6349 PET/CT.

^68^Ga-MY6349 PET/CT detected 92 additional distant metastatic lesions compared with ^18^F-FDG PET/CT, including 58 bone lesions, 14 liver lesions, eight lung lesions, two splenic lesions, four brain lesions, and six lesions at uncommon metastatic sites, such as the pleura, ovaries, and peritoneum. Overall, among 600 malignant and 49 benign lesions, ^68^Ga-MY6349 PET/CT correctly identified 564 of 600 malignant lesions, with only two false-positive findings. The characteristics of the 36 false-negative lesions are summarized in Table S7.

Among the 564 true-positive lesions detected by ^68^Ga-MY6349 PET/CT, 123 (21.8%) were histologically confirmed, whereas the remaining were verified through clinical and radiologic follow-up. All imaging-confirmed lesions underwent follow-up for a minimum of 6 months. The median follow-up duration was 6 (range, 6–9) months.

In contrast, only 436/600 true positives were identified via ^18^F-FDG PET/CT, and the rate of false positives was higher, with 40 benign lesions exhibiting increased ^18^F-FDG uptake. These results suggest that ^68^Ga-MY6349 PET/CT offers superior diagnostic accuracy in the identification of malignant lesions than ^18^F-FDG PET/CT.

### ^68^Ga-MY6349 PET/CT Leads to a Higher Rate of Changes in Clinical Management Compared with ^18^F-FDG PET/CT

We compared TNM classification and staging between the PET/CT modalities for the 30 patients with newly diagnosed BC. Notably, the primary breast tumors were not identified via ^18^F-FDG PET/CT in five patients, whereas multiple lesions were successfully detected via ^68^Ga-MY6349 PET/CT in all five patients, resulting in T-stage upgrades in four patients from Tx to T1 and in one patient from Tx to T2. Additionally, further primary breast lesions were detected in three other patients with ^68^Ga-MY6349 PET/CT, with two, two, and one extra lesions, respectively. However, the T classification was not altered for these patients (Figure 2A).

**Figure 3:**
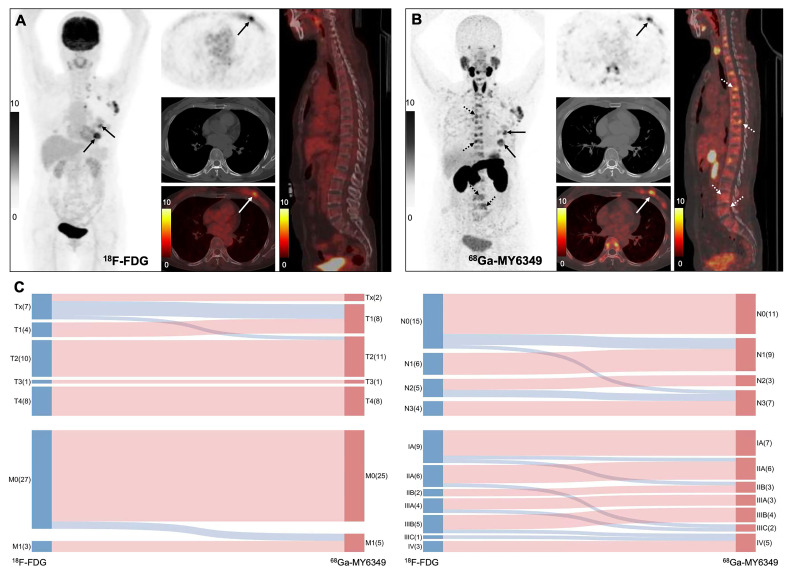
Impact of ^68^Ga-MY6349 PET/CT on clinical staging. (**A, B**) An 80-year-old woman presented with a painful mass in the left breast that had persisted for >1 month. Histopathology confirmed a diagnosis of micropapillary carcinoma, classified as luminal B HER2-positive cancer. Both ^18^F-FDG and ^68^Ga-MY6349 PET/CT revealed a high uptake in the primary lesion and axillary lymph node metastases. However, multiple vertebral lesions with heterogeneous bone density had negative uptakes upon ^18^F-FDG PET/CT but positive uptakes upon ^68^Ga-MY6349 PET/CT. As a result, the clinical stage was revised from T2N3M0 (based on ^18^F-FDG) to T2N3M1 (based on ^68^Ga-MY6349). (**C**) Sankey diagram illustrating changes in TNM staging among 30 newly diagnosed patients, based on ^18^F-FDG PET/CT vs. ^68^Ga-MY6349 PET/CT. ^18^F-FDG: ^18^F-fluorodeoxyglucose; CT: computed tomography; HER2: epidermal growth factor receptor 2; PET: positron emission tomography.

Significant differences were observed regarding N-staging results between the PET/CT modalities. With ^18^F-FDG PET/CT, the distribution of N stages was: 50% N0, 20% N1, 17% N2, and 13% N3. In contrast, ^68^Ga-MY6349 PET/CT yielded a distribution of 37% N0, 30% N1, 10% N2, and 23% N3, resulting in an overall N-stage upstaging rate of 20% (Figure 2B). In terms of M staging, two patients classified at M0 with ^18^F-FDG PET/CT were reclassified at M1 with ^68^Ga-MY6349 PET/CT owing to the identification of newly detected distant bone metastases (Figure 3A, 3B).

In total, compared with ^18^F-FDG PET/CT, ^68^Ga-MY6349 PET/CT resulted in upgrades in TNM staging in 6/30 newly diagnosed patients (20%; Table S6, Figure 3C), including upgrades from stage IA to IIA (n = 1), IA to IIB (n = 1), IIA to IIIC (n = 1), IIIA to IIIC (n = 1), IIIB to IV (n = 1), and IIIC to IV (n = 1). Consequently, treatment strategies were adjusted for four of the six patients. In two cases, clinical management transitioned from initial surgical intervention to neoadjuvant therapy, followed by surgery. In the remaining two cases, the approach shifted from neoadjuvant therapy to systemic palliative chemotherapy.

**Figure 4:**
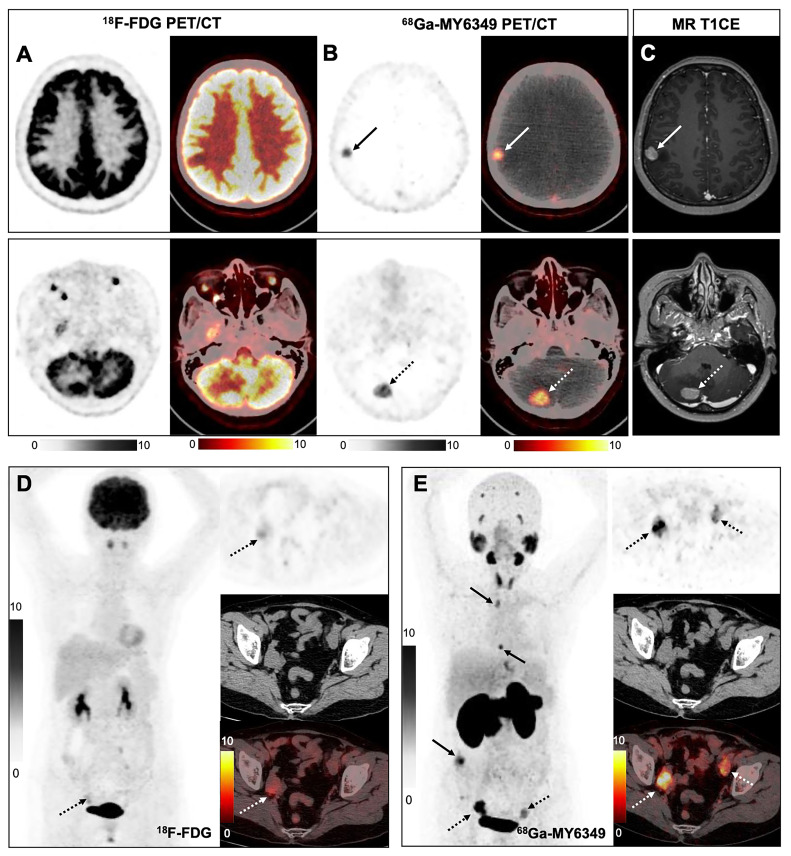
Use of ^68^Ga-MY6349 PET/CT for breast-cancer restaging. (**A–C**) A 43-year-old woman presented with a persistent headache 5 months after surgery for left breast lobular carcinoma (luminal B, HER2^-^). PET/CT was performed to evaluate possible recurrence or metastasis. Owing to the intrinsically high physiological background in the brain, ^18^F-FDG PET/CT failed to clearly identify any brain lesions (**A**). In contrast, ^68^Ga-MY6349 PET/CT detected a metastatic lesion in the right frontoparietal lobe (**B**). Subsequent MRI confirmed the ^68^Ga-MY6349 PET/CT features. (**D, E**) Another 43-year-old woman presented with progressively rising CEA levels 4 years after surgery for left breast lobular carcinoma (luminal B, HER2^-^). ^18^F-FDG PET/CT showed no definitive hypermetabolic lesions; a mild uptake was observed in the right adnexal region, difficult to distinguish from physiological ovarian activity (**D**). In contrast, ^68^Ga-MY6349 PET/CT revealed bilateral adnexal metastases (**E**). Additionally, ^68^Ga-MY6349 PET/CT revealed metastases in the mediastinal lymph nodes, thoracic spine, and mesentery not visualized with ^18^F-FDG PET/CT. Based on these observations, both patients’ treatment plans were revised from routine surveillance to systemic chemotherapy. ^18^F-FDG: ^18^F-fluorodeoxyglucose; CT: computed tomography; HER2: epidermal growth factor receptor 2; MRI: magnetic resonance imaging; PET: positron emission tomography.

Among the 43 patients who underwent PET/CT for restaging, an additional 100 lesions were detected across 15 patients (15/43, 35%) via ^68^Ga-MY6349 PET/CT compared with ^18^F-FDG PET/CT, leading to change in the treatment regimen for five patients (12%). Notably, in three of these cases, ^18^F-FDG PET/CT led to false-negative results, whereas ^68^Ga-MY6349 PET/CT revealed metastases in the brain, bones, and ovaries (Figure 4), leading from active surveillance to systemic chemotherapy. In the remaining two cases, ^18^F-FDG PET/CT produced false-positive findings (bone and lymph node lesions), whereas ^68^Ga-MY6349 PET/CT correctly classified these lesions as benign, thereby preventing unnecessary overtreatment (Figure S1-2).

**Figure 5:**
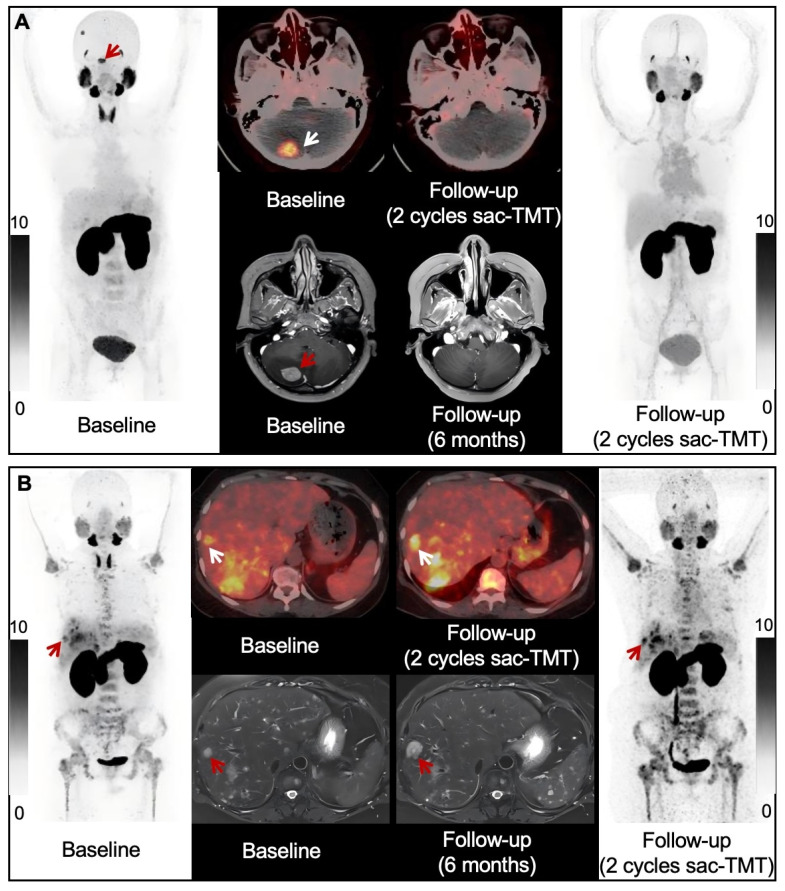
Changes in ^68^Ga-MY6349 uptake as a potential predictor of response to Trop2-ADC. (**A**) A 43-year-old woman with metastatic TNBC. Compared with the baseline scan, follow-up ^68^Ga-MY6349 PET/CT (two cycles after sac-TMT treatment) revealed significant decrease in ^68^Ga-MY6349 uptake in the brain metastases. The follow-up MRI (6 months after treatment) revealed complete response, with disappearance of the brain lesions. (**B**) A 46-year-old woman with metastatic TNBC. Compared to the baseline scan, follow-up ^68^Ga-MY6349 PET/CT (two cycles after sac-TMT treatment) revealed higher ^68^Ga-MY6349 uptake in most liver and bone metastases. The follow-up MRI (6 months after treatment) revealed progressive disease. ADC: antibody-drug conjugate; CT: computed tomography; MRI: magnetic resonance imaging; PET: positron emission tomography; sac-TMT: sacituzumab tirumotecan; TNBC: triple-negative breast cancer; Trop2: trophoblast cell-surface antigen 2.

At our center, another prospective study evaluating ^68^Ga-MY6349 PET/CT for Trop2 ADC response assessment in BC (NCT07046689) is currently ongoing. To date, three patients with TNBC have completed a preliminary 6-month follow-up. At baseline, all three patients demonstrated positive tracer uptake in tumor lesions on PET imaging. However, after two treatment cycles, the patients exhibited markedly different PET responses. In one patient, tracer uptake in brain metastases decreased markedly following treatment (SUV_max_ decreased from 8.2 to undetectable; Figure 5A), and a complete response was subsequently achieved at the 6-month follow-up. In contrast, another patient demonstrated substantially increased tracer uptake after two cycles of therapy (representative SUV_max_ increased from 6.6 to 10.5; Figure 5B). At the 6-month follow-up, this patient developed progressive disease, with the representative lesion increasing in size from 1.4 cm to 2.4 cm. The remaining patient exhibited disease progression accompanied by increased ^68^Ga-MY6349 uptake, consistent with the pattern observed in the representative progressive case.

### Correlation Between PET/CT Parameters and Clinicopathological Features

**Figure 6:**
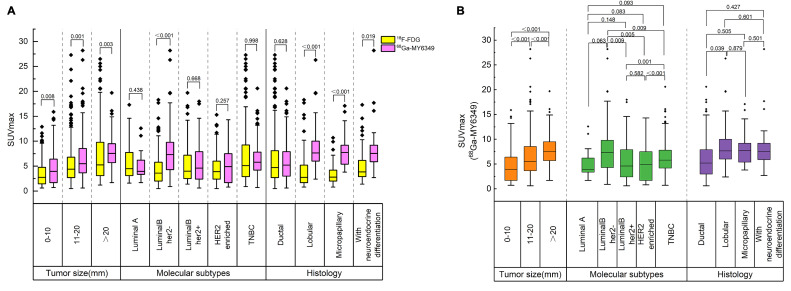
Box plots of PET parameters across 600 lesions stratified according to tumor pathology. (**A**) Comparison of SUV_max_ between ^68^Ga-MY6349 and ^18^F-FDG PET/CT, displayed as box plots according to tumor size, receptor status—including HR, HER2, and TNBC—and histological subtype. (**B**) Subgroup analysis of ^68^Ga-MY6349 SUV_max_, presented as box plots categorized by the same pathological features as those in (**A**). Group comparisons for continuous variables were conducted using the Mann–Whitney *U* test or independent-samples *t*-test, depending on the normality of the data distribution. A two-sided P-value <0.05 was considered statistically significant. HER2^-^, HER2-negative; HER2^+^, HER2-positive; HR^+^, HR-positive. ^18^F-FDG: ^18^F-fluorodeoxyglucose; CT: computed tomography; HER2: epidermal growth factor receptor 2; HR: hormone receptor; PET: positron emission tomography; SUVmax: maximum standardized uptake value; TNBC: triple-negative breast cancer.

^68^Ga-MY6349 PET/CT yielded higher SUV_max_ values than ^18^F-FDG PET/CT across all tumor size categories: small (quartile [Q]1–Q3: 1.7–6.4 vs. 1.4–4.8, P = 0.008), medium (Q1–Q3: 3.6–8.6 vs. 2.7–6.8, P = 0.001), and large (Q1–Q3: 5.7–9.5 vs. 3.1–9.8, P = 0.003). When stratified according to molecular subtype, a significantly higher uptake of ^68^Ga-MY6349 vs. ^18^F-FDG was only observed in luminal B HER2-negative tumors (Q1–Q3: 4.3–9.8 vs. 2.0–5.8, P < 0.001). From a histopathological perspective, tumor uptake was significantly higher with ^68^Ga-MY6349 than with ^18^F-FDG in tumors with lobular histology (P < 0.001), micropapillary features (P < 0.001), and neuroendocrine differentiation (P = 0.019, Figure 6A). When grouped by molecular subtype, the ^68^Ga-MY6349 PET/CT SUV_max_ showed a descending trend across the following groups: luminal B HER2-negative, TNBC, HER2-positive, luminal B HER2-positive, and luminal A. However, not all intergroup differences were significant. Furthermore, among the four histological subtypes analyzed—ductal, lobular, micropapillary, and neuroendocrine—no significant differences were observed regarding the ^68^Ga-MY6349 PET/CT SUV_max_, except between the ductal and lobular subtypes (Figure 6B).

## Discussion

Trop2 has emerged as a pivotal biomarker in clinical oncology, characterized by its frequent overexpression across a wide spectrum of epithelial tumors 8, 9. This study examined the utility of ^68^Ga-MY6349 PET/CT for BC diagnosis and treatment decisions, given the prominent expression of Trop2 in breast malignancies 18 and validation of ^68^Ga-MY6349 PET/CT for *in vivo* Trop2 imaging 15.

We found that ^68^Ga-MY6349 PET/CT offers clinical advantages for BC diagnosis and staging over standard imaging modalities. ^68^Ga-MY6349 PET/CT exhibited superior detection of primary tumors than both breast ultrasound and ^18^F-FDG PET/CT, enabling exhaustive preoperative lesion mapping, which is critical for surgical planning and lesion characterization, particularly for multifocal and invasive carcinomas that may otherwise be missed. ^68^Ga-MY6349 PET/CT yielded higher tumor uptake and superior imaging contrast for regional lymph-node staging, resulting in greater diagnostic accuracy in N-staging, directly influencing treatment planning. Unlike ^18^F-FDG, known to yield false-positives owing to increased inflammatory or reactive lymph-node uptake 19, ^68^Ga-MY6349 PET/CT achieved high specificity and a high nodal-lesion detection rate. The markedly lower physiological uptake of ^68^Ga-MY6349 in normal brain tissue translated into substantially higher lesion-to-background contrast than that with ^18^F-FDG, which could improve brain-metastasis detection. Although Trop2 is expressed in certain normal epithelial tissues, it is frequently overexpressed in epithelial malignancies, including BC 20. Therefore, physiological Trop2 expression is unlikely to substantially compromise the diagnostic specificity of ^68^Ga-MY6349. The notably enhanced diagnostic performance, especially in TNBC and luminal B HER2-negative BC, suggests that ^68^Ga-MY6349 PET/CT may play a crucial role in detecting these tumor subtypes, whose accurate nodal staging profoundly influences treatment choices.

Although ^18^F-FDG PET/CT is the standard-of-care imaging for BC distant-metastasis evaluation 21, here, ^68^Ga-MY6349 PET/CT outperformed ^18^F-FDG PET/CT, particularly in the detection of lymph node, bone, visceral, and brain metastases. ^18^F-FDG uptake reflects glucose metabolism and cellularity 22, but it often underperforms in detecting sclerotic bone lesions 22, 23. Additionally, physiological ^18^F-FDG uptake in cerebral gray matter significantly reduces lesion-to-background contrast, limiting ^18^F-FDG PET/CT sensitivity for intracranial-metastasis detection 24, 25. Conversely, ^68^Ga-MY6349 PET/CT, which is not influenced by bone remodeling and exhibits minimal background activity in the brain, allows clearer visualization of such lesions. Herein, the use of ^68^Ga-MY6349 PET/CT facilitated the detection of 128 metastatic lesions not identified with ^18^F-FDG PET/CT, providing critical information and allowing more precise tumor staging and modifications to treatment strategies for approximately 15% of patients. From a clinical management perspective, the superior diagnostic performance of ^68^Ga-MY6349 PET/CT may lead to stage migration in a substantial proportion of patients, particularly through detecting previously occult nodal and distant metastases. Such refined staging may directly alter treatment decisions: patients who are upstaged to stage IV may shift from curative-intent locoregional therapies to systemic treatment options, whereas those with a more precise disease-extent characterization may benefit from more appropriately tailored radiation fields or surgical strategies. Nevertheless, ^68^Ga-MY6349 PET/CT failed to detect 36 malignant lesions. The missed lesions were generally small and may have been affected by partial-volume effects. In addition, intertumoral heterogeneity in Trop2 expression may have contributed to reduced tracer uptake in some lesions.

^68^Ga-MY6349 uptake consistently exceeded that of ^18^F-FDG when stratified according to tumor size and histological subtype. When stratified according to molecular subtype, the highest SUV_max_ was observed in luminal B HER2-negative BC, followed by TNBC. These findings are consistent with current clinical indications for Trop2-targeted ADC use, especially in TNBC and luminal B HER2-negative subtypes 12-14, 26-28. Therefore, ^68^Ga-MY6349 PET/CT may hold particular clinical relevance for those BC subtypes. ^68^Ga-MY6349 PET/CT’s superior imaging contrast and quantitative accuracy enables more precise TNM staging and recurrence monitoring than conventional ^18^F-FDG PET/CT. Thus, ^68^Ga-MY6349 PET/CT may critically assist personalized treatment planning and selection of patients most likely to benefit from Trop2-targeted therapies, providing a non-invasive approach to evaluate target engagement and treatment response.

Although baseline Trop2 expression is essential for the target binding of ADCs, it may be insufficient to predict the treatment response by itself, which is influenced by additional factors, including drug internalization and payload release. This is corroborated by findings from the phase III ASCENT trial, which revealed no association between Trop2 expression (assessed by H-score quartiles) and overall survival benefit (P=0.054) in metastatic TNBC 26. Similarly, in luminal B HER2-negative BC, sacituzumab govitecan has exhibited comparable efficacy, regardless of baseline Trop2 expression levels (median overall survival: 14.6 months for an H-score <100 vs. 14.4 months for an H-score ≥100) 27. Although both TNBC and luminal B HER2-negative tumors typically express Trop2, baseline expression alone may not suffice to distinguish responders from non-responders. An imaging technique capturing early treatment-induced changes in tumor activity may better assess therapeutic efficacy. Therefore, we investigated whether ^68^Ga-MY6349 PET/CT could predict the response to Trop2-targeted ADC therapy.

Although patient enrollment is ongoing, preliminary observations from three representative TNBC cases suggest that early changes in SUV_max_ on Trop2 PET after two cycles of Trop2-ADC may reflect treatment response. Marked reduction in lesion SUV_max_ was associated with favorable clinical outcomes, whereas stable or increased uptake was associated with disease progression. These exploratory findings from TNBC cases suggest that early changes in SUV_max_ may be associated with treatment response, warranting further prospective validation in larger patient populations. Consistent with this observation, a recent study of nectin-4–targeted ADC therapy demonstrated that reduction >30% in tracer uptake after treatment was associated with improved therapeutic efficacy 29. Collectively, these data support further ^68^Ga-MY6349 PET/CT prospective evaluation for disease characterization and early treatment-response monitoring in BC.

Certain study limitations should be acknowledged. First, although 73 patients were enrolled, the number of cases within each molecular subtype was relatively small, precluding robust subtype-specific analyses. Studies with larger, more balanced cohorts should validate the findings across distinct BC subtypes. Second, as ^18^F-FDG uptake is not tumor-specific and may be influenced by various physiologic and pathologic conditions, residual confounding in lesion-uptake assessment cannot be entirely excluded despite standardized imaging procedures. Third, lesion-level correlations between SUV_max_ and Trop2 H-scores were not evaluated, as the relationship between ^68^Ga-MY6349 uptake and Trop2 expression had already been systematically established 15. Fourth, only three patients with TNBC treated with Trop2-targeted ADC therapy had available 6-month follow-up data, reflecting the exploratory nature of this analysis. Ongoing enrollment is expected to allow more rigorous evaluation and validation of these findings. Despite these limitations, the present results highlight the potential clinical utility of serial ^68^Ga-MY6349 PET/CT in guiding Trop2-targeted therapy.

## Conclusions

^68^Ga-MY6349 PET/CT was superior to ^18^F-FDG PET/CT for primary and metastatic BC diagnosis, with important implications for treatment planning and clinical decision-making. These findings support the integration of ^68^Ga-MY6349 PET/CT in routine practice to improve diagnostic accuracy and guide personalized treatment strategies toward precision medicine.

## Supplementary Material

Supplementary figures and tables.

## Figures and Tables

**Table 1:** Characteristics of patients with confirmed breast cancer

Characteristics	n
Number	73
Age, median (range)	52 (29–86)
Sex	
Female	70
Male	3
Clinical indication for PET/CT	
Initial staging	30
Recurrence/restaging	43
Histology	
Invasive breast carcinoma, no special type	
Ductal	55
With neuroendocrine differentiation	4
Special invasive types	
Lobular	10
Micropapillary	2
Apocrine adenocarcinoma	1
Mucinous adenocarcinoma	1
Molecular subtypes	
Luminal A	7
Luminal B	38
HER2^+^	(8)
HER2^-^	(30)
HER2-positive	9
TNBC	19

CT: computed tomography; HER2: human epidermal growth factor receptor 2; PET: positron emission tomography; TNBC, triple-negative breast cancer.

**Table 2:** Comparison of ^18^F-FDG and ^68^Ga-MY6349 uptake in primary and metastatic breast cancer

Tumor location	^18^F-FDG PET/CT		^68^Ga-MY6349 PET/CT		P^1^	
	SUV_max_, median (Q1-Q3)	TBR, median (Q1-Q3)	SUV_max_, median (Q1-Q3)	TBR, median (Q1-Q3)	SUV_max_ (MY6349 vs. FDG)	TBR (MY6349 vs. FDG)
Breast lesions	4.1 (2.7–7.3)	2.9 (1.3–4.8)	4.9 (3.8–7.2)	6.6 (3.3–11.2)	0.163	< 0.001
Local/distant lymph node metastases	4.2 (2.4–7.0)	5.3 (3.0–9.6)	5.6 (3.5–8.1)	7.8 (4.5–12.9)	0.002	< 0.001
Bone and visceral metastases	4.0 (2.6–6.8)	3.0 (1.8–5.2)	6.3 (3.6–8.8)	5.0 (2.6–7.1)	< 0.001	< 0.001
Brain metastases	15.3 (10.2–18.3)	1.0 (0.8–1.0)	8.5 (5.8–12.2)	6.1 (4.3–23.7)	0.063	0.018
Uncommon metastases^2^	3.8 (2.1–8.1)	3.4 (1.6–6.5)	6.4 (2.9–9.0)	6.3 (3.8–7.9)	0.268	0.046
Total	4.1 (2.5–7.2)	3.4 (2.1–6.7)	5.9 (3.6–8.6)	6.0 (3.6–9.6)	< 0.001	< 0.001

CT: computed tomography; PET: positron emission tomography; FDG: fluorodeoxyglucose; Q: quartile; SUV_max_: maximum standardized uptake value; TBR: tumor-to-background ratio. 1: Wilcoxon signed-rank test. 2: Uncommon metastases include muscular, subcutaneous, gingival, ovarian, pleural and mesenteric metastases.

**Table 3:** Lesion-based comparison of ^18^F-FDG PET/CT and ^68^Ga-MY6349 PET/CT for the diagnosis of primary and metastatic breast cancer

Site of disease	New malignant lesions^1^ (FDG/MY6349)	Coherent malignant lesions	Benign lesions	Total lesions	
				^18^F-FDG PET/CT	^68^Ga-MY6349 PET/CT
Primary/recurrent tumors	1/10	49	7	50 TPLs, 10 FNLs, 7 TNLs, 0 FPLs	59 TPLs, 1 FNL, 7 TNLs, 0 FPLs
Regional/distant lymph node metastases	8/55	158	36	166 TPLs, 58 FNLs, 0 TNLs, 36 FPLs	213 TPLs, 11 FNLs, 36 TNLs, 0 FPLs
Bone and visceral metastases	19/82	173	5	192 TPLs, 86 FNLs, 2 TNLs, 3 FPLs	255 TPLs, 23 FNLs, 3 TNLs, 2 FPLs
Brain metastases	0/4	3	0	3 TPLs, 4 FNLs, 0 TNLs, 0 FPLs	7 TPLs, 0 FNLs, 0 TNLs, 0 FPLs
Uncommon metastases	1/6	24	1	25 TPLs, 6 FNLs, 0 TNLs, 1 FPL	30 TPLs, 1 FNL 1 TNL, 0 FPLs
Total	29/157	407	49	436 TPLs, 164 FNLs, 9 TNLs, 40 FPLs	564 TPLs, 36 FNLs, 47 TNLs, 2 FPLs

1: New malignant lesions, additional malignant lesions detected by one tracer that were not identified by the other tracer. FDG, additional lesions identified via ^18^F-FDG PET/CT compared with ^68^Ga-MY6349 PET/CT; MY6349, additional lesions identified via ^68^Ga-MY6349 PET/CT compared with ^18^F-FDG PET/CT. ^18^F-FDG: ^18^F-fluorodeoxyglucose; CT: computed tomography; FNL: false-negative lesion; FPL: false-positive lesion; PET: positron emission tomography; TNL: true-negative lesion; TPL: true-positive lesion

## Data Availability

The data that support the findings of this study are available from the corresponding author upon reasonable request.

## Abbreviations

^18^F-FDG: ^18^F-fluorodeoxyglucose
ADC: antibody-drug conjugate
BI-RADS: Breast Imaging Reporting and Data System
CT: computed tomography
HER2: human epidermal growth factor receptor 2
MRI: magnetic resonance imaging
PET: positron emission tomography
SUVmax: maximum standardized uptake value
TBR: tumor-to-background ratio
TNBC: triple-negative breast cancer
Trop2: trophoblast cell-surface antigen 2
