# Supplementary material for: Trop2-targeted PET/CT imaging with 68Ga-MY6349 improves diagnostic accuracy and influences clinical management in breast cancer
Source: Theranostics. 2026 Jul 13;16(14):8117–28. doi: 10.7150/thno.136704 (PMC13440513; doi:10.7150/thno.136704)
Supplement: Supplementary file 1 [file thnov16p8117s1.pdf]

Supplementary materials

The PET/CT scanning and image reconstruction protocols

Radiotracer doses were adjusted according to body weight: 3.7 MBq/kg for ^{18}F-FDG and 1.8–2.2 MBq/kg for ^{68}Ga-MY6349. Patients fasted for at least 6 h prior to ^{18}F-FDG imaging to ensure normal blood glucose levels. No fasting or specific preparation was required before ^{68}Ga-MY6349 PET/CT. PET/CT was initiated 60 min after radiotracer injection by using a hybrid scanner (Discovery MI; GE Healthcare, Milwaukee, WI, USA). All scans were performed according to the protocol as we have previously described (reference [36] in the manuscript). Briefly, a PET scan was immediately performed after the CT scan in 3-dimensional acquisition mode with 6–8 bed positions and 2.0–2.5 min/position. All the obtained data were transferred to the Advantage Workstation (version AW 4.7, GE Healthcare); data were reconstructed using the Bayesian penalized likelihood (BPL) reconstruction algorithm (Q.clear, GE Healthcare), with a penalization factor (beta) of 500. Then, the reconstructed images were co-registered and displayed.

Supplementary Figures

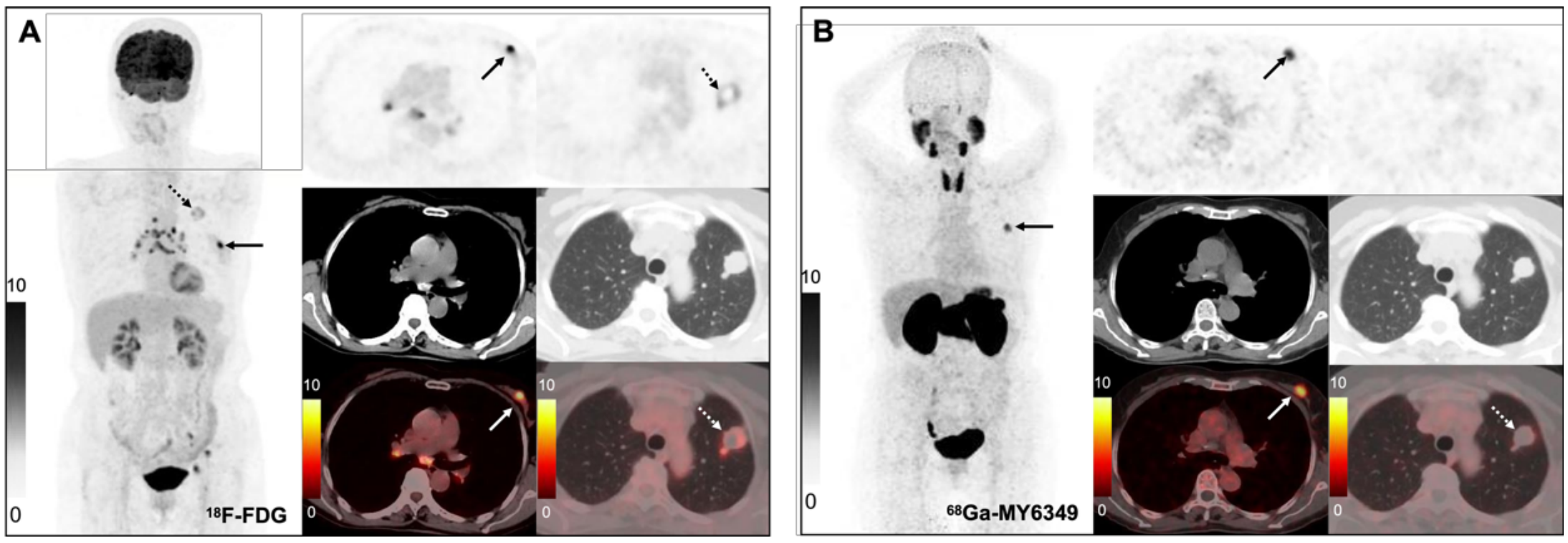

Figure S1. Representative cases demonstrating false-positive findings upon ^{18}F-FDG PET/CT but correctly diagnosed via ^{68}Ga-MY6349 PET/CT. (**A**) A 67-year-old woman presented with a suspicious left breast nodule and underwent PET/CT for preoperative staging. ^{18}F-FDG PET/CT revealed multiple hypermetabolic lymph nodes in the bilateral hilar, mediastinal, pelvic, and left inguinal regions, along with a hypermetabolic soft-tissue nodule in the left upper lobe of the lung. (**B**) Upon subsequent ^{68}Ga-MY6349 PET/CT, all of these nodes and nodules were negative for tracer uptake, suggestive of a benign etiology. Histopathological analysis of the nodule in the left lung revealed the presence of benign proliferating myofibroblasts. The patient later underwent breast surgery, and follow-up imaging at 6 months revealed no progression in the lymph nodes or pulmonary lesion, further supporting their benign nature.

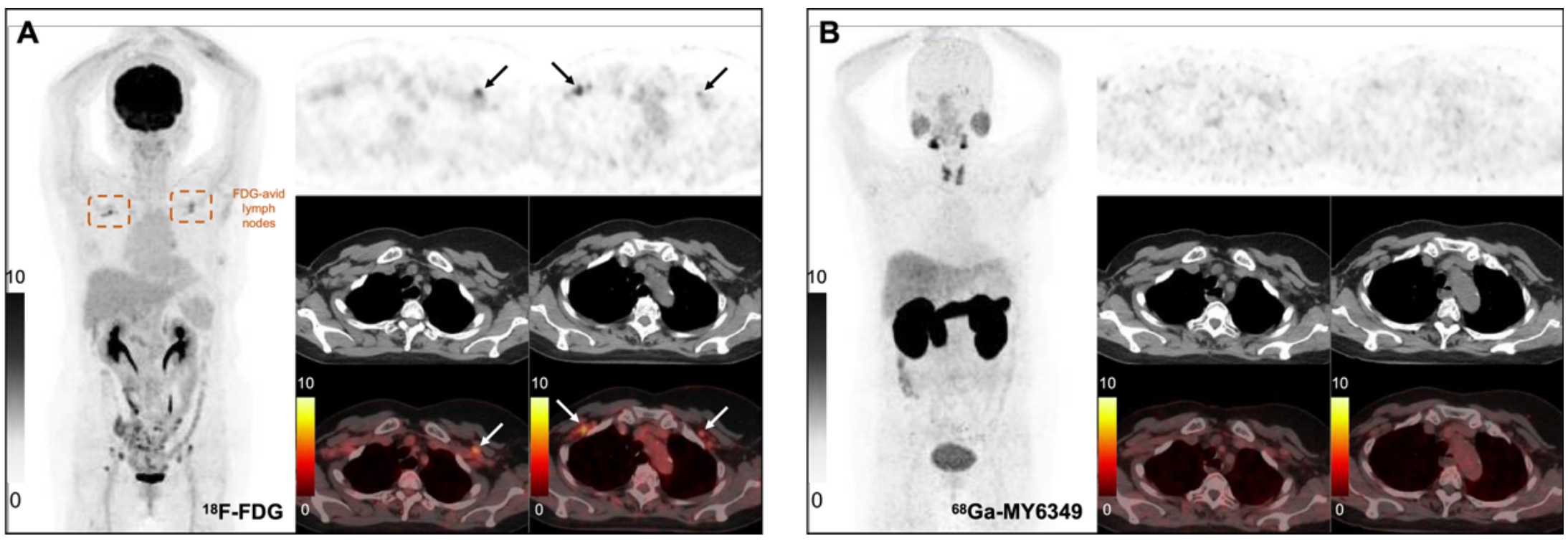

Figure S2. Representative case demonstrating the value of ^{68}Ga-MY6349 PET/CT in avoiding necessary treatment. (**A**) A 62-year-old woman with a history of right breast cancer presented with multiple enlarged lymph nodes in the chest-wall intermuscular spaces upon routine follow-up CT 4 years after surgery. ^{18}F-FDG PET/CT revealed a high bilateral uptake in the chest wall and inguinal lymph nodes (SUV_{max}, 2.7–8.1; TBR, 2.5–7.4), suggestive of disease recurrence. (**B**) In contrast, ^{68}Ga-MY6349 PET/CT revealed a negative uptake in the corresponding lymph nodes (SUV_{max}, 1.0–2.1; TBR, 0.6–1.2), suggesting a benign etiology. Subsequent follow-up CT at 6 months confirmed resolution of the lymphadenopathy, consistent with inflammatory rather than malignant lesions.

Supplementary Tables

Table S1. Comparison of ^{18}F-FDG and ^{68}Ga-MY6349 uptake in Luminal A breast cancer

Tumor location	^{18}F-FDG PET/CT	^{68}Ga-MY6349 PET/CT	P*

	SUV_{max} (Median, IQR)	TBR (Median, IQR)	SUV_{max} (Median, IQR)	TBR (Median, IQR)	SUV_{max} (MY6349 vs. FDG)	TBR (MY6349 vs. FDG)
Breast lesions	2.9 (1.7–4.2)	1.9 (1.1–2.8)	3.9 (3.3–11.1)	5.1 (3.0-–5.9)	0.063	**0.018**
Local/distant lymph node metastases	6.3 (4.6–9.2)	8.5 (6.1–12.4)	4.4 (4.0–6.0)	5.9 (4.4–7.0)	0.109	0.285
Bone and visceral metastases	8.6 (4.2–12.3)	5.1 (3.1–8.5)	3.6 (3.2–5.2)	2.7 (2.2–4.4)	**0.046**	**0.028**
Brain metastases	NA	NA	NA	NA	NA	NA
Uncommon metastases†	NA	NA	NA	NA	NA	NA
Total	4.5 (3.0–8.5)	3.3 (2.2–8.1)	4.0 (3.3–6.3)	4.1 (2.6–6.7)	0.438	0.796

Table S2. Comparison of ^{18}F-FDG and ^{68}Ga-MY6349 uptake in Luminal B HER2^{-} breast cancer

Tumor location	^{18}F-FDG PET/CT		^{68}Ga-MY6349 PET/CT		P*	
	SUV_{max} (Median, IQR)	TBR (Median, IQR)	SUV_{max} (Median, IQR)	TBR (Median, IQR)	SUV_{max} (MY6349 vs. FDG)	TBR (MY6349 vs. FDG)
Breast lesions	4.7 (2.6–7.3)	2.7 (1.1–4.4)	4.5 (3.7–6.1)	3.8 (2.3–8.3)	0.808	**0.003**
Local/distant lymph node metastases	2.5 (1.6–3.5)	3.1 (1.8–4.6)	6.4 (2.6–10.3)	11.0 (3.2–17.0)	**<0.001**	**<0.001**
Bone and visceral metastases	4.4 (2.7–6.2)	2.7 (1.8–4.2)	7.9 (5.6–9.5)	5.8 (3.9–7.7)	**<0.001**	**<0.001**
Brain metastases	NA	NA	NA	NA	NA	NA
Uncommon metastases†	3.3 (1.5–7.1)	2.8 (1.5–3.9)	9.0 (7.9–10.5)	6.3 (4.6–7.9)	**0.001**	**0.001**
Total	3.6	3.0	7.3	6.1	**<0.001**	**<0.001**

	(2.0–5.8)	(1.6–4.2)	(4.2–9.8)	(3.5–10.2)

Table S3. Comparison of ^{18}F-FDG and ^{68}Ga-MY6349 uptake in Luminal B HER2^{+} breast cancer

Tumor location	^{18}F-FDG PET/CT		^{68}Ga-MY6349 PET/CT		P*	
	SUV_{max} (Median, IQR)	TBR (Median, IQR)	SUV_{max} (Median, IQR)	TBR (Median, IQR)	SUV_{max} (MY6349 vs. FDG)	TBR (MY6349 vs. FDG)
Breast lesions	3.7 (2.9–5.7)	3.4 (1.4–4.3)	4.9 (3.9–8.8)	7.8 (3.8–13.4)	0.003	**<0.001**
Local/distant lymph node metastases	4.8 (3.3–9.4)	7.9 (3.7–11.7)	4.6 (3.0–8.8)	6.8 (4.5–10.6)	0.912	0.422
Bone and visceral metastases	3.1 (2.4–7.1)	2.6 (1.8–4.9)	3.4 (1.8–7.1)	2.0 (1.3–4.7)	0.686	0.717
Brain metastases	NA	NA	NA	NA	NA	NA
Uncommon metastases†	NA	NA	NA	NA	NA	NA
Total	4.0 (2.5–7.5)	3.7 (2.5–6.7)	4.6 (2.4–8.0)	4.8 (2.3–8.2)	0.668	0.128

Table S4. Comparison of ^{18}F-FDG and ^{68}Ga-MY6349 uptake in HER2-positive breast cancer

Tumor location	^{18}F-FDG PET/CT		^{68}Ga-MY6349 PET/CT		P*	
	SUV_{max} (Median, IQR)	TBR (Median, IQR)	SUV_{max} (Median, IQR)	TBR (Median, IQR)	SUV_{max} (MY6349 vs. FDG)	TBR (MY6349 vs. FDG)
Breast lesions	6.0 (3.2–10.0)	5.5 (2.7–6.6)	3.9 (2.4–6.0)	3.3 (1.8–8.5)	0.611	0.176
Local/distant lymph node metastases	5.5 (2.7–7.2)	8.3 (3.3–10.6)	4.9 (4.0–8.3)	5.4 (4.4–11.3)	0.199	0.923
Bone and visceral metastases	3.5 (1.4–5.2)	2.8 (1.1–7.6)	1.7 (1.4–5.4)	2.3 (1.7–5.8)	0.925	0.958
Brain metastases	NA	NA	NA	NA	NA	NA
Uncommon metastases†	2.1 (1.7–3.1)	1.8 (1.4–2.6)	5.9 (4.0–7.6)	11.1 (7.5–14.3)	**0.042**	**0.043**
Total	3.9	3.3	4.9	5.5	0.257	0.194

	(2.4–6.0)	(1.8–8.5)	(1.7–7.5)	(2.3–8.5)	

Table S5. Comparison of ^{18}F-FDG and ^{68}Ga-MY6349 uptake in TNBC

Tumor location	^{18}F-FDG PET/CT		^{68}Ga-MY6349 PET/CT		*P**	
	SUV_{max} (Median, IQR)	TBR (Median, IQR)	SUV_{max} (Median, IQR)	TBR (Median, IQR)	SUV_{max} (MY6349 vs. FDG)	TBR (MY6349 vs. FDG)
Breast lesions	4.7 (2.2–13.5)	2.9 (1.4–7.5)	5.5 (3.3–7.2)	10.4 (4.4–12.4)	0.101	**0.010**
Local/distant lymph node metastases	5.5 (2.8–9.8)	6.8 (3.7–13.6)	5.2 (3.8–6.4)	8.3 (5.0–12.5)	0.327	**0.034**
Bone and visceral metastases	4.0 (2.9–8.8)	3.3 (2.0–6.3)	6.6 (5.2–8.7)	5.3 (4.1–7.4)	**0.005**	**<0.001**
Brain metastases	NA	NA	NA	NA	NA	NA
Uncommon metastases†	7.9 (6.2–11.6)	7.1 (5.6–10.5)	2.4 (1.9–4.2)	3.6 (3.0–4.5)	**0.003**	**0.013**
Total	5.1 (2.9–9.3)	4.8 (2.6–8.4)	5.8 (4.2–7.8)	6.4 (4.3–	0.988	**<0.001**

	10.2)

Table S6. Comparative analysis of TNM classifications via ^{18}F-FDG PET/CT vs. ^{68}Ga-MY6349 PET/CT in patients with newly diagnosed breast cancer

Category	Stage	^{18}F-FDG PET/CT	^{68}Ga-MY6349 PET/CT	Upstaging
T (n=30)	T1	4	8	5
	T2	10	11	
	T3	1	1	
	T4	8	8	
	Tx	7	2	
N (n=30)	N0	15	11	6
	N1	6	9	
	N2	5	3	
	N3	4	7	
M (n=30)	M0	27	25	2
	M1	3	5	
TNM staging system (n=30)	IA	9	7	6
	IIA	6	6	
	IIB	2	3	
	IIIA	4	3	
	IIIB	5	4	

IIIC	1	2
IV	3	5

Table S7. Characteristics of the 36 false-negative lesions on ^{68}Ga-MY6349 PET/CT

Characteristic	Value
Lesion location, n (%)	
Breast lesions	1 (2.8%)
Lymph node metastases	11 (30.6%)
Bone and visceral metastases	23 (63.9%)
Uncommon metastases	1 (2.8%)
Imaging features	
^{68}Ga-MY6349 SUV_{max}, median (IQR)	1.4 (1.0–2.2)
Size, median (IQR), mm	11 (8-14)
Histology, n (%)	
Ductal	36 (100.0%)
Molecular subtypes	
Luminal A	1 (2.8%)
Luminal B HER2-negative	7 (19.4%)
Luminal B HER2-positive	26 (72.2%)
TNBC	2 (5.6%)
